# Cracking the Cytotoxicity Code: Apoptotic Induction of 10-Acetylirciformonin B is Mediated through ROS Generation and Mitochondrial Dysfunction

**DOI:** 10.3390/md12053072

**Published:** 2014-05-22

**Authors:** Huei-Chuan Shih, Mohamed El-Shazly, Yung-Shun Juan, Chao-Yuan Chang, Jui-Hsin Su, Yu-Cheng Chen, Shou-Ping Shih, Huei-Mei Chen, Yang-Chang Wu, Mei-Chin Lu

**Affiliations:** 1 Department of Nursing, Meiho University, Pingtung 912, Taiwan; E-Mail: x00002213@meiho.edu.tw; 2 Department of Pharmacognosy and Natural Products Chemistry, Faculty of Pharmacy, Ain-Shams University, Organization of African Unity Street, Abassia, Cairo 11566, Egypt; E-Mail: elshazly444@googlemail.com; 3 Department of Urology, Kaohsiung Municipal Hsiao-Kang Hospital, Kaohsiung 812, Taiwan; E-Mail: juanuro@gmail.com; 4 Department of Urology, College of Medicine, Kaohsiung Medical University, Kaohsiung 807, Taiwan; 5 Department of Urology, Kaohsiung Medical University Hospital, Kaohsiung 807, Taiwan; 6 Department of Anatomy, College of Medicine, Kaohsiung Medical University, Kaohsiung 807, Taiwan; E-Mail:chaoyuah@kmu.edu.tw; 7 Graduate Institute of Marine Biotechnology, National Dong Hwa University, Pingtung 944, Taiwan; E-Mails: x2219@nmmba.gov.tw (J.-H.S.); j520c@hotmail.com (Y.-C.C.); m6430005@hotmail.com (S.-P.S.); 8 National Museum of Marine Biology & Aquarium, Pingtung 944, Taiwan; 9 Pingtung Branch of Kaohsiung Veterans General Hospital, Nutrition Branch, Pingtung 912, Taiwan; E-Mail: amy.chenhm@msa.hinet.net; 10 Natural Medicinal Products Research Center, China Medical University Hospital, Taichung 404, Taiwan; 11 Center for Molecular Medicine, China Medical University Hospital, Taichung 404, Taiwan

**Keywords:** 10-acetylirciformonin B, apoptosis, hexokinase, mitochondria, reactive oxygen species (ROS), topoisomerase

## Abstract

A marine furanoterpenoid derivative, 10-acetylirciformonin B (10AB), was found to inhibit the proliferation of leukemia, hepatoma, and colon cancer cell lines, with selective and significant potency against leukemia cells. It induced DNA damage and apoptosis in leukemia HL 60 cells. To fully understand the mechanism behind the 10AB apoptotic induction against HL 60 cells, we extended our previous findings and further explored the precise molecular targets of 10AB. We found that the use of 10AB increased apoptosis by 8.9%–87.6% and caused disruption of mitochondrial membrane potential (MMP) by 15.2%–95.2% in a dose-dependent manner, as demonstrated by annexin-V/PI and JC-1 staining assays, respectively. Moreover, our findings indicated that the pretreatment of HL 60 cells with *N*-acetyl-l-cysteine (NAC), a reactive oxygen species (ROS) scavenger, diminished MMP disruption and apoptosis induced by 10AB, suggesting that ROS overproduction plays a crucial rule in the cytotoxic activity of 10AB. The results of a cell-free system assay indicated that 10AB could act as a topoisomerase catalytic inhibitor through the inhibition of topoisomerase IIα. On the protein level, the expression of the anti-apoptotic proteins *Bcl-xL* and *Bcl-2*, caspase inhibitors XIAP and survivin, as well as hexokinase II were inhibited by the use of 10AB. On the other hand, the expression of the pro-apoptotic protein Bax was increased after 10AB treatment. Taken together, our results suggest that 10AB-induced apoptosis is mediated through the overproduction of ROS and the disruption of mitochondrial metabolism.

## 1. Introduction

The oncogene revolution in the last three decades has rekindled interest in studying survival-related pathways in cancer cells with the ultimate goal of developing efficient anticancer therapeutics targeting these pathways [1]. Much focus has been directed on studying mitochondria and their role in cancer cell survival-related pathways. Mitochondria are the principal energy factories in living cells, which play crucial roles in the cellular survival pathway. Thus, they have become major targets in chemotherapy-induced apoptosis against cancer cells [2,3,4]. One of the most vital functions of mitochondria is energy production in the form of ATP. In normal differentiated cells, the bulk of ATP is produced in mitochondria through the process of oxidative phosphorylation (OXPHOS) [5]. However under stressful conditions, OXPHOS is usually suppressed. Cancer cells need to evade any process limiting energy supply, especially in the tight abnormal tumor microenvironment and thus they shift from OXPHOS to glycolysis, aiming to produce sufficient ATP. This effect is one of the cancer phenotype hallmarks and known as “Warburg effect” [6]. It allows cancer cells to produce ATP at a faster rate compared with OXPHOS, despite the overall lower efficiency. Recent studies have suggested that hexokinase is a key enzyme that catalyzes the first step in the glycolysis pathway. This enzyme transfers a phosphate group from ATP to glucose to form glucose-6-phosphate [7]. In cancer tissues, the high glycolytic activity requires an up-regulation of the key glycolytic enzymes, including hexokinase. Interestingly, the percentage of hexokinase binding to mitochondria significantly increases in cancer cells [8,9,10]. Targeting the interplay between mitochondrial hexokinase and cancer cells can provide potential opportunities for the development of new anticancer drugs [11].

Another molecular mechanism that has recently caught significant attention in the continuous war on cancer is the relationship between the accumulation of reactive oxygen species (ROS) and mitochondrial dysfunction [12]. Accumulating evidence suggested that ROS-regulated apoptosis is a promising target for anticancer drugs [13,14]. The manipulation of ROS can influence cancer cell survival, growth, and differentiation. Therefore, targeting the associated pathways can act as an attractive therapeutic strategy against cancer. Recent findings have suggested that the overproduction of ROS can induce apoptosis in cancer cells [15,16]. Moreover, it was found that the anti-apoptotic effect of Bcl-2, which can be related to certain cases of anticancer drug resistance, acts by decreasing the overall cellular oxidative stress through the suppression of the NADPH oxidase complex [17,18]. This protein plays a pivotal role in maintaining mitochondrial integrity and function, as well as in regulating the oxidative metabolic machinery [19]. Additionally, it has been observed that the tumor suppressor gene p53 might induce apoptosis through the induction of ROS production and the upregulation of Bax and PUMA [20]. Thus, inducing ROS generation is expected to increase the sensitivity of cancer cells to apoptotic stimuli in cancer therapy. With these targets in mind, our group has implemented an ambitious plan to explore and elaborate the detailed mechanism of action for some newly isolated cytotoxic compounds from marine organisms. Defining the molecular targets for biologically active compounds is a crucial step in developing any pharmaceutical drug, because it provides the necessary information for the development of highly specialized *in vivo* models and accurate future clinical trials [21,22].

In our previous report, we identified a series of cytotoxic C_21_ and C_22_ terpenoid-derived metabolites in the ethyl acetate (EtOAc) extract from the marine sponge, *Ircinia* sp. [23]. Among the isolates, 10-acetylirciformonin B (10AB) exhibited the highest cytotoxic activity [23]. The potent activity of 10AB encouraged us to investigate the underlying mechanism of action. The cytotoxic evaluation of 10AB against several cancer cell lines revealed that the most vulnerable cell line was HL 60 [24]. Our preliminary results suggested that the 10AB cytotoxic effect against HL 60 cells is mediated through DNA damage and apoptotic induction [24]. Aiming to further investigate the cytotoxic mechanism of 10AB, we examined the effect of 10AB on topoisomerase IIα, mitochondrial stability, and ROS generation in the HL 60 cancer cell line.

## 2. Results

### 2.1. The Apoptotic Induction Effect of 10AB in HL 60 Cells

**Figure 1:**
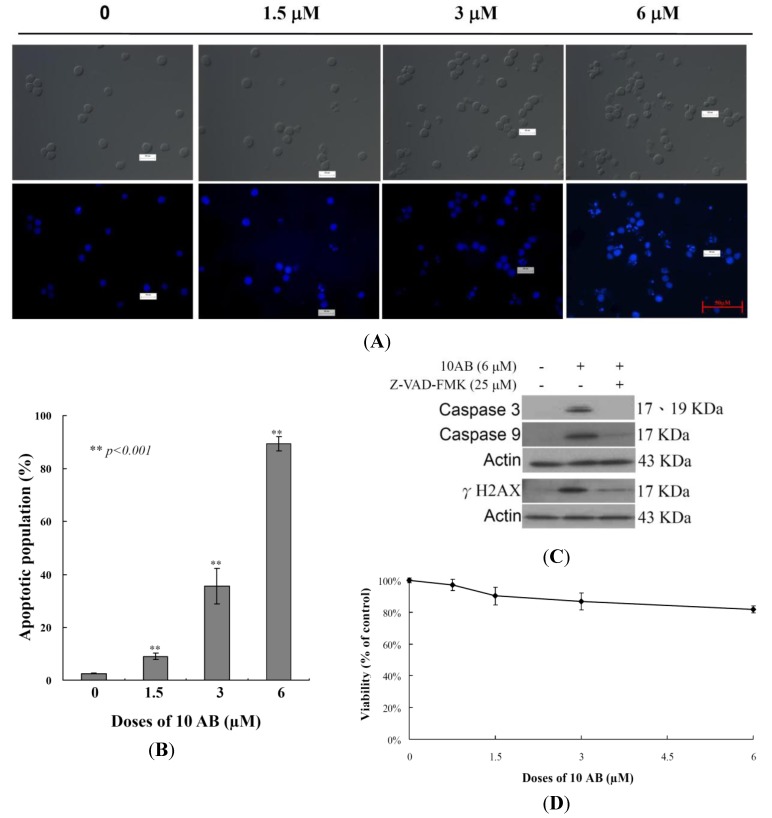
A furanoterpenoid derivative, 10AB, induces apoptosis in HL 60 cells. The cells were treated with different doses of 10AB (0, 1.5, 3.0, and 6.0 μM) for 24 h. (**A**) The treated cells were stained with DAPI. The morphological changes were examined with fluorescence microscopy; (**B**) The treated cells were stained with annexin-V/PI and examined with flow cytometry. The results are presented as means ± SD of three independent experiments and ** *p* < 0.001 indicats statistically significant differences compared with the control group (DMSO treatment); (**C**) Cells were pretreated with or without 25 μM of Z-VAD-FMK and then treated with 6.0 μM of 10AB for 24 h. Cell lysates were analyzed via immunoblotting with specific antibodies; (**D**) The viability of normal rat alveolar macrophage NR8383 cells was determined with different doses of 10AB (0, 1.5, 3.0, and 6.0 μM).

The anti-proliferative and apoptotic induction effects of 10AB in HL 60 cells were demonstrated in our previous report [24]. However, to set the stage for a deeper investigation of the 10AB apoptotic mechanism, it was necessary to further confirm this effect in the current study. The effect of 10AB on nuclear morphology was evaluated utilizing DAPI staining. As shown in Figure 1A, the control group cell nuclei were large and round; however, the nuclei of the treated cells were fragmented and condensed, suggesting that the cells suffered from apoptotic induction. We also analyzed how increasing 10AB concentrations affected the HL 60 apoptotic population utilizing flow cytometric assessment. As shown in Figure 1B, the use of 10AB (1.5, 3.0 and 6.0 μM) resulted in a remarkable increase in the percentage of apoptotic cells (8.9% ± 1.2%, 35.6% ± 2.1%, 87.6% ± 3.47%, respectively) in comparison with the control group (2.5% ± 0.2%). These results confirmed that 10AB suppressed cancer cell growth through apoptotic induction. In our previous study, we demonstrated that the pretreatment of HL 60 cells with caspase 8 or 9 inhibitors attenuated the effect of 10AB by 13% and 27%, respectively [24]. In the current work, we further examined the relationship between caspases and the apoptotic effect induced by 10AB. Caspases 3 and 9 activation was significantly diminished with the pretreatment of a pan caspase inhibitor, Z-VAD-FMK, as confirmed by Western blotting. Additionally, pretreatment with the pan caspase inhibitor slightly diminished γH2AX induction by 10AB (Figure 1C). These results suggested that the apoptotic effect of 10AB is partially mediated through the caspase pathway. To determine whether the cytotoxic effect of 10AB is specific for cancer cells, we examined the effect of 10AB on the viability of rat alveolar macrophage NR8383 cells. Even at the highest dose (6.0 μM), 10AB treatment caused only 18.3% suppression in the viability of NR8383 cells (Figure 1D). Thus, it may be concluded that 10AB’s cytotoxic effect is more specific towards HL 60 cells compared to normal macrophage NR8383 cells.

### 2.2. The Effect of 10AB on Topoisomerase IIα Activity

**Figure 2:**
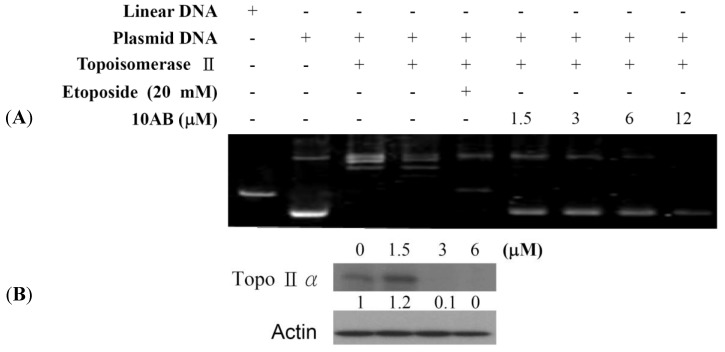
The effect of 10AB on topo IIα function. (**A**) The effect of 10AB on topo IIα mediated supercoiled pHOT1 plasmid DNA relaxation with cell-free system; (**B**) 10AB decreased the expression of topo IIα protein in HL 60 cells. HL 60 cells were treated with 10AB (0, 1.5, 3.0 and 6.0 μM) for 24 h. The protein expression of topo IIα was analyzed via Western blotting. The bands were quantified via densitometry and normalized relative to β-actin levels.

Our previous work showed that 10AB treatment could induce DNA damage in HL 60 cells as deduced from the abnormal tail size in the comet assay and the increase in H2AX phosphorylation (γH2AX) [24]. To further determine if the DNA damaging effect is associated with the interruption of topoisomerase II (topo II) activity, we utilized cell-free DNA cleavage assay using an enzyme-mediated negatively supercoiled pHOT1 plasmid DNA (Figure 2A). Lane 1 shows a linear DNA strand, which was also observed upon treating the supercoiled pHOT1 plasmid DNA with etoposide, a standard topo II poison (Lane 5) [25,26]. The use of 10AB in increasing concentrations (0, 1.5, 3.0, 6.0 and 12.0 μM) inhibited DNA relaxation and resulted in the formation of supercoiled DNA products in the presence of topo IIα (Lanes 6–9). Moreover, we observed an inhibitory effect of 10AB on topo IIα protein expression, which plays a critical role in DNA replication, transcription and chromosomal segregation [27]. Western blotting indicated that the use of 10AB (3.0 μM) significantly diminished topo IIα protein expression (Figure 2B). These results suggested that one of 10AB’s targets as a DNA damaging agent is to interfere with certain steps of the topo IIα catalytic cycle.

### 2.3. The Relationship between 10AB-Induced Apoptosis in HL 60 Cells and the Disruption in Mitochondrial Membrane Potential as well as Mitochondrial Metabolism-Related Proteins

**Figure 3:**
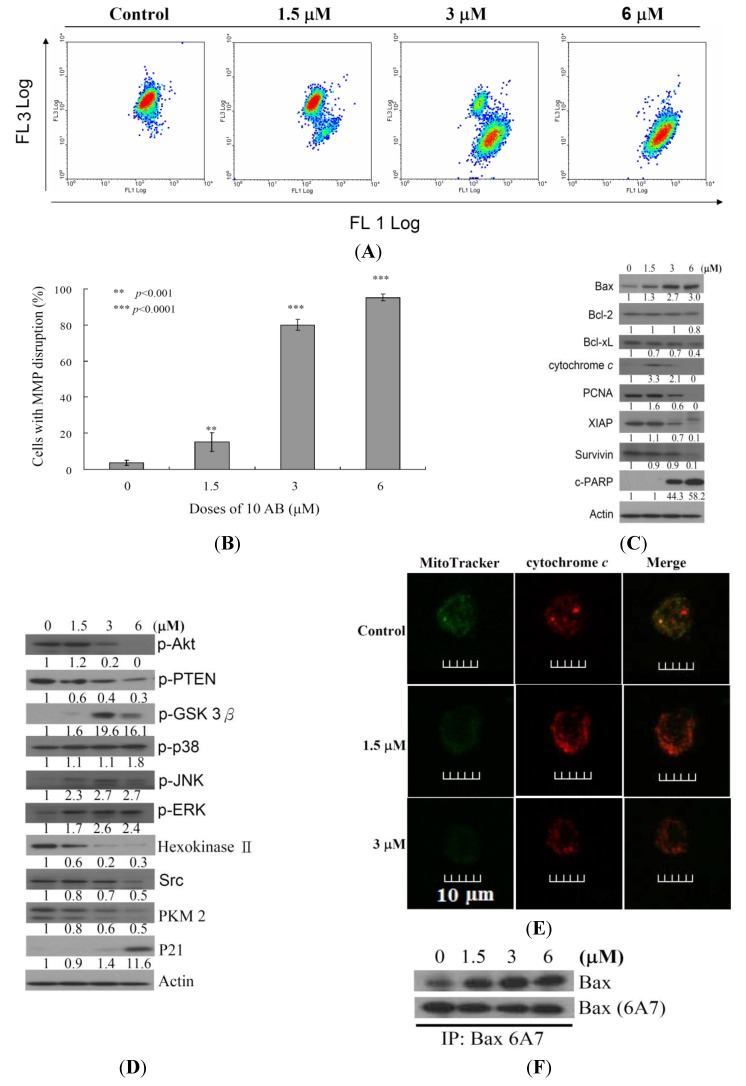
Apoptotic induction induced by 10AB involves mitochondrial dysfunction. The effect of 10AB treatment on the mitochondrial membrane potential (MMP) in HL 60 cells was evaluated. (**A**,**B**) Cells were treated with 10AB (1.5, 3.0, and 6.0 μM) for 24 h. Quantitative results showed a significant increase in HL 60 cells with disturbed MMP in response to the use of 10AB. Results are presented as means ± SD of three independent experiments (** *p* < 0.01; *** *p* < 0.001). The effect of 10AB on the expression of apoptotic; (**C**) and mitochondrial metabolism; (**D**) related proteins in HL 60 cells. The bands were quantified via densitometry and normalized to β-actin levels; (**E**) Merged images of Mitotracker with cytochrome *c* immunofluorescence suggested that the increase in cytochrome *c* was the direct effect of 10AB treatment at 1.5 of 3 μM; (**F**) Cell lysates were subjected to immunoprecipitation with anti-Bax6A7 antibody and analyzed by immunoblotting with anti-Bax antibody.

After confirming the apoptotic effect of 10AB on HL 60 cells, it was necessary to determine the apoptotic inducer within these cells. We previously demonstrated that treating HL 60 cells with 10AB resulted in caspases 3 and 9 activation [24]. In the current work, we examined if the dysfunction in proteins related to mitochondrial membrane potential and mitochondrial metabolism is involved in 10AB apoptotic induction. JC-1 cationic dye was used to evaluate whether 10AB treatment influenced the mitochondrial membrane potential (MMP) in HL 60 cells. As shown in Figure 3A,B, the use of 10AB (1.5 μM) increased the population of HL 60 cells with disrupted membrane potential from 3.6% to 15.2%. This effect was dramatically increased upon treatment with 10AB at 3.0 and 6.0 μM, which resulted in 80.0% ± 3.0% and 95.2% ± 1.8% cells with disturbed MMP, respectively (** *p* < 0.001). To further explore the mechanism of 10AB-induced apoptosis, the effect of 10AB on apoptotic- and mitochondrial metabolism-related proteins was evaluated. As shown in Figure 3C, 10AB treatment suppressed the anti-apoptotic proteins (Bcl-2 and Bcl-xL), caspase inhibitor (XIAP), and survivin. Concomitantly, 10AB treatment increased the dysfunction of DNA repair genes (PARP cleavage), cytochrome *c* concentration, and the pro-apoptotic protein Bax. Moreover, treatment of leukemia HL 60 cells with different concentrations of 10AB diminished the expression of p-Akt (Ser^473^), p-PTEN (Ser^38^^0^), Src, hexokinase II, and PKM 2, but enhanced the expression of p-ERK, p-38, p-JNK, and p-GSK 3β(Ser^9^) (Figure 3D). In agreement with the results of Western blotting experiments, images from confocal scanning laser microscopy demonstrated that treatment with 10AB (1.5 or 3.0 μM) was able to enhance the fluorescence of cytochrome *c*. When HL 60 cells were treated with DMSO for 24 h, cytochrome *c* was largely localized in the mitochondria, as revealed by the yellow-orange staining in the overlay. This indicated that green fluorescence (MitoTracker Green) derived from mitochondria and the cytochrome *c*-associated red fluorescence co-localized. Treatment with 10AB (1.5 or 3.0 μM) resulted in the release of cytochrome *c* from the mitochondria into the cytosol, as demonstrated by the appearance of the red fluorescence in the cytoplasm (Figure 3E). This cytochrome *c*-associated red fluorescence was considerably more discernible in HL 60 cells treated with 3.0 μM 10AB than in the control group. These results indicated that 10AB treatment could release cytochrome *c* in HL 60 cells. To further elucidate the apoptotic induction mechanism, we examined the effect of 10AB treatment on Bax conformational change. Previous studies indicated that conformational change of Bax could be induced by several types of apoptotic stimuli [28]. This conformational change can be detected with anti-Bax6A7, which recognizes only the activation/pro-apoptotic form of Bax [29]. We conducted an immunoprecipitation experiment to determine if 10AB treatment could enhance the expression of the active form of Bax. As shown in Figure 3F, 10AB treatment increased the active form of Bax, suggesting that apoptosis induced by 10AB involves Bax activation.

### 2.4. The Relationship between 10AB-Induced Apoptosis and ROS Generation

**Figure 4:**
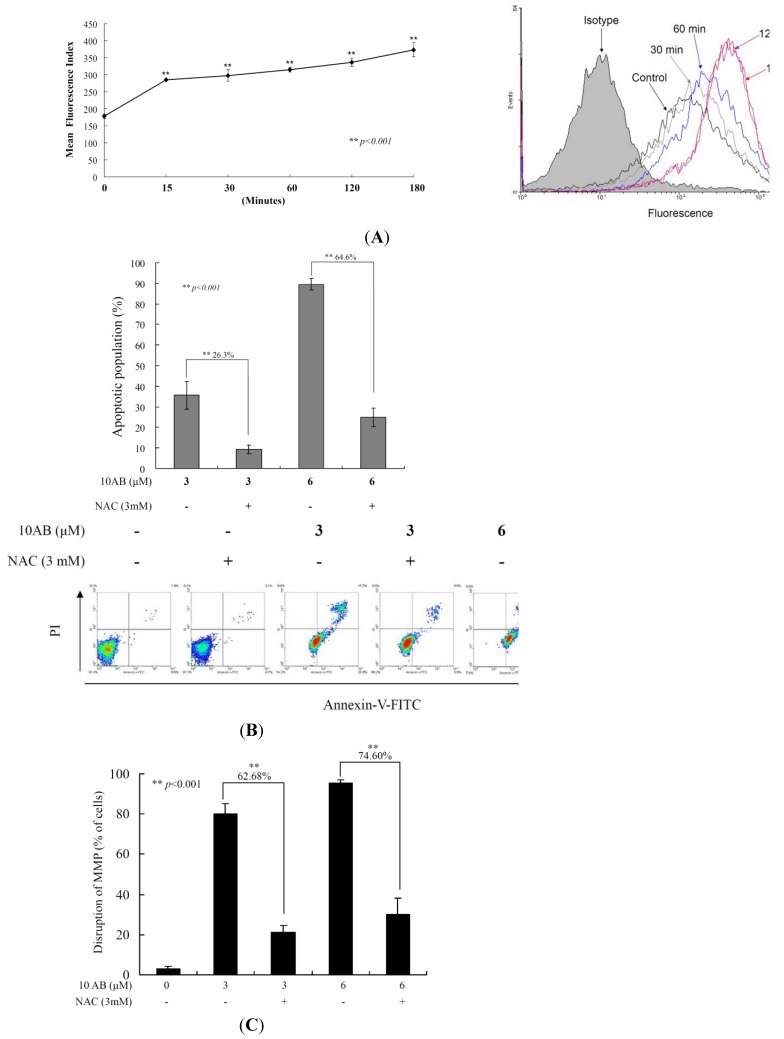
The apoptotic induction of 10AB in HL 60 cells involves ROS production. We evaluated the effect of the 10AB treatment on ROS generation in HL 60 cells. (**A**) Cells were treated with 10AB (6.0 μM) for the indicated times. Quantitative results showed a gradual increase in the ROS production in response to the 10AB treatment when compared with the control group. We also evaluated the effect of ROS generation on the 10AB-induced apoptosis in HL 60 cells. Cells were pretreated with 3.0 mM NAC for 2 h, then treated with 3.0 or 6.0 μM of 10AB; The apoptotic populations (**B**) and the disruption of MMP (**C**) were examined with annexin-V/PI and JC-1 staining assay. Results are presented as mean ± SD of three independent experiments (** *p* < 0.001).

To examine whether the 10AB-induced apoptosis in HL 60 cells involves the overproduction of ROS, we determined the levels of ROS at different times following 10AB treatment. A time-dependent increase in ROS generation was monitored using the carboxy derivative of fluorescein, carboxy-H_2_DCFDA dye. As shown in Figure 4A, 10AB treatment (3.0 μM) for 15, 30, 60, 120 and 180 min resulted in 1.60-, 1.67-, 1.77-, 1.89- and 2.1-fold increase in the ROS levels, respectively, as compared with the mean fluorescence index (MFI) of the control. To clarify whether ROS generation is the major regulator in 10AB-induced apoptosis, HL 60 cells were pretreated with NAC, a ROS scavenger, aiming to suppress the intracellular oxidative stress. The apoptotic population was measured via annexin V/PI staining following 10AB treatment. As shown in Figure 4B, the result of NAC treatment is similar to the negative control group showing less than 5% of the apoptotic population. In addition, NAC pretreatment diminished the apoptotic cell population from 35.6% and 89.5% to 9.3% and 25% in response to the use of 3.0 and 6.0 μM of 10AB, respectively. These results indicated that blocking oxidative stress by NAC resulted in saving HL 60 from apoptosis induced by 10AB. To further confirm if MMP disruption induced by 10AB is initiated by ROS overproduction, we determined the population of cells with disturbed MMP in response to 10AB treatment with or without NAC pretreatment. The determination of the cell population with disturbed MMP was achieved utilizing a cationic dye, JC-1 (Figure 4C).Cells were divided into four groups, in which two groups were only treated with 3.0 and 6.0 μM of 10AB, whereas the other two groups were treated with NAC (3.0 mM) followed by 3.0 or 6.0 μM of 10AB. After 24 h, the change in the population of cells with disturbed MMP was analyzed in the four groups. The NAC pretreatment diminished the population of cells with disturbed MMP from 80.0% and 95.3% to 17.4% and 20.7% in response to the treatment with 3 and 6 μM of 10AB, respectively. In agreement with the preceding results of annexin V/PI staining, these findings indicate that the cytotoxic effect of 10AB in HL 60 is mediated through apoptotic induction as well as mitochondrial dysfunction and this effect is highly influenced by ROS production.

## 3. Discussion

Our previous work suggested that the natural marine furanoterpenoid derivative, 10AB, exhibited potent cytotoxic activity in several cancer cell lines, including DLD-1, Hep G2 and 3B, and K 562 cells [23,24]. It was found that the antiproliferative activity of 10AB was mediated through the induction of DNA damage and apoptosis in leukemia HL 60 cells [24]. The DNA damaging effect of 10AB was demonstrated by the induction of H2AX phosphorylation (γH2AX) and the increase of tail movement in the comet assay, suggesting increased DSBs [24]. In the current study, we tried to uncover other regulatory mechanisms involved in 10AB-induced apoptosis. Our results indicated that the induction of γH2AX caused by 10AB was slightly diminished by the pretreatment with a pan caspase inhibitor, Z-VAD-FMK (Figure 1C). These findings suggested that the apoptotic effect of 10AB was partially mediated through the caspase pathway. This effect was also observed in certain cytotoxic drugs that induce apoptosis, partially through the caspase-mediated pathway [30]. Furthermore, we studied the effect of 10AB on DNA topoisomerase II, ROS generation, MMP, and apoptotic- and mitochondrial metabolism-related proteins. DNA topoisomerase II inhibitors have emerged as a promising class of cancer chemotherapeutics, targeting cancer cells through the induction of DSBs [31]. Etoposide, doxorubicin, and mitoxantrone are clinically approved anticancer drugs, which are known as the first class of DNA topoisomerase II inhibitors. It was found that these drugs can stabilize a cleavage complex in DNA, leading to topo II mediated chromosome DNA breakage [27,32]. Because these agents generate a “lesion” that includes DNA strand breaks and proteins covalently bound to DNA, they have been known as topo II poisons. Unfortunately, in certain cases, the use of these agents has resulted in serious drawbacks, including the induction of secondary malignancies [33,34,35,36]. On the other hand, the second class of DNA topo II inhibitors only diminished the enzymatic activity of topo II proteins [27]. To study the effect of 10AB on topo II, we utilized a cell-free DNA cleavage assay. It was found that even at the lowest concentration, 10AB completely inhibited the formation of DNA relaxation (Figure 2A). These results were further confirmed through the elimination of topo II protein expression in response to 10AB (3.0 and 6.0 μM) treatment, as shown in Figure 2B. These findings indicated that the apoptotic effect of 10AB was partially mediated through the inhibition of topo II activity in human acute promyelocytic leukemia HL 60 cells. Unlike etoposide, 10AB can be developed as a topo II catalytic inhibitor, avoiding the side effects of topo II poisons.

Another factor in the cellular survival mechanism that has recently attracted attention is the proliferating cell nuclear antigen (PCNA). It is a nuclear factor protein and a proliferation marker involved in the repair of proliferating cells, DNA replication, and in the regulation of cellular survival pathways [37]. PCNA and other proteins, including p21, cdc/cyclin B1, and MAPKs, are stress response kinases, which are involved in cell cycle regulation. Recent studies have suggested that Gadd45 proteins, which act as stress sensors, are mediated by a complex interplay of physical interactions with stress response kinases [38,39]. Cells that are deficient in Gadd45 proteins are more sensitive to radio- and chemotherapy (ultraviolet radiation, VP-16, and daunorubicin) induced apoptosis compared to wild-type (wt) cells [39]. Interaction of Gadd45 with PCNA or histone or both might play a role in epigenetic gene activation through DNA repair-mediated demethylation [40]. Although cancer cells have the capability to repair DNA damage, extensive fragmentation can lead to the activation of apoptotic triggers [41]. Our results suggested that the elimination of PCNA expression and the induction of PARP dysfunction were the major contributors to 10AB-induced DNA damage in HL 60 cells (Figure 3C).

Moving to another target in the apoptotic pathway, we investigated the interplay between mitochondria and apoptosis [42]. Previous reports have indicated that chemotherapeutic induction of mitochondrial oxidative stress results in the activation of GSK and Bax, phosphorylation of its chaperone cyclophilin D, and in the facilitation of permeability transition poreopening [43]. Furthermore, a conformational change as a consequence of the exposure of 6A7 epitope in the *N*-terminus (amino acids 13–19) has been suggested to activate the pro-apoptotic function of Bax [44]. The active form of Bax (Bax mutant with the 6A7 epitope conformational change) can be inserted efficiently into the mitochondrial membrane, resulting in cytochrome *c* release, which is one of the key steps in the apoptotic induction mediated by caspase activation in mammalian cells. A previous investigation has suggested that intracellular ROS production is an upstream factor for Bax activation and cytochrome *c* release [45]. In 2011, McCubrey *et al*. [46] proposed that the Ras/Raf/mitogen-activated protein kinase (MEK)/extracellular signal-regulated kinase (ERK) pathway contributes to the sensitivity and resistance of leukemia cells in response to chemotherapy. It was found that the Ras/Raf/MEK/ERK pathway can be activated by chemotherapeutic drugs commonly used in leukemia therapy. The potent cytotoxic activity of 10AB against leukemia cancer cells has encouraged us to investigate its effect on the Ras/Raf/MEK/ERK pathway. We found that 10AB at 3.0 and 6.0 μM resulted in an increase in the percentage of apoptotic cells with perturbed MMP reaching 80.0% and 95.2%, respectively. As shown in Figure 3C,D, 10AB treatment up-regulated Bax activation and cytochrome *c* release as well as ERK, p-38, JNK, and GSK 3β. Concomitantly, 10AB down-regulated the phosphorylation of PTEN and Akt. Based on these findings, it is clear that 10AB targets the Ras/Raf/MEK/ERK and PI3K/PTEN/Akt/mTOR pathways, which implies a potential to block the survival pathways and induce the apoptotic pathways.

Our results were in agreement with previous reports suggesting that the increase in the release of cytochrome *c*, GSK phosphorylation at Ser 9, and the induction of Bax conformational change are the major contributors to the mitochondrial collapse in cancer cells induced by 10AB [42,43,44,45,46]. As outlined above, 10AB treatment elevated intracellular oxidative stress, which interrupted mitochondrial metabolism and triggered apoptosis in HL 60 cells. Based on the Warburg effect, cancer cells entirely reprogram their metabolism to sustain hyperproliferation and mostly rely on glycolysis rather than oxidative phosphorylation for ATP production [47]. Hexokinases (HK) are over-expressed in many tumor cells, contributing to the enhanced tumor capacity for oxidative glycolysis and apoptosis suppression [48,49]. Notably, these proteins are the rate-limiting glycolytic enzymes in the irreversible first step of the glycolysis [49,50,51]. Gall *et al.* proposed that the loss of mitochondrial HK II is clearly associated with mitochondrial Bax accumulation, AIF release, and caspase 3 activation, resulting in apoptotic cell death [3]. Our findings were consistent with previous reports suggesting that the elimination of proteins related to mitochondrial metabolism, such as HK II, PKM 2, and Src, participates in the 10AB-induced mitochondrial dysfunction via oxidative stress.

Our work suggests that 10AB is a potent cytotoxic agent against acute myelocytic leukemia, which is one of the most common and progressive malignancies among malignant hematopoiesis. Several chemotherapeutic agents have been developed that target this aggressive disease with mediocre results. The need for additional chemotherapeutics is becoming increasingly vital; however, the number of the newly developed candidates has been on the wane. Recently, the clinical application of all-*trans* retinoic acid in acute promyelocytic leukemia, which targets the effect of peroxiredoxins as a defensive mechanism in cancer cells against oxidative stress, has offered mixed results [52]. We think that the activity of 10AB against specific molecular targets in HL 60 cells offers a promising alternative to be further developed and optimized as a potential candidate for the treatment of acute myelocytic leukemia.

## 4. Experimental Section

### 4.1. Bioassays Materials

HL 60 (human promyelocytic leukemia) cells were obtained from the American Type Culture Collection (ATCC, Manassas, VA, USA). Cells were maintained in RPMI 1640 medium supplemented with 10% fetal calf serum, 2 mM glutamine, and antibiotics (100 units/mL penicillin and 100 μg/mL streptomycin) at 37 °C in a humidified atmosphere of 5% CO_2_. RPMI 1640 medium, fetal calf serum (FCS), trypan blue, penicillin G, and streptomycin were obtained from Gibco BRL (Gaithersburg, MD, USA). Dimethyl sulfoxide (DMSO), 3-(4,5-dimethylthiazol-2-yl)-2,5-diphenyl-tetrazolium bromide (MTT), and all other chemicals were purchased from Sigma-Aldrich (St. Louis, MO, USA). Antibodies against c-PARP, p-PTEN (Ser^38^^0^), p-GSK 3β (Ser^9^), p-Akt (Ser^473^), hexokinase, p-ERK, p-p38, p-JNK, PCNA, PKM2, and Src were purchased from Cell Signaling Technologies (Beverly, MA, USA). Antibodies for Bax, Bcl-2, Bcl-xL, cytochrome *c*, survivin, topoisomerase IIα, and XIAP were obtained from Santa Cruz Biotechnology (Santa Cruz, CA, USA). JC-1 cationic dye and the carboxy derivative of fluorescein (carboxy-H_2_DCFDA) were purchased from Molecular Probes and Invitrogen technologies (Carlsbad, CA, USA). Anti-mouse and rabbit IgG peroxidase-conjugated secondary antibody were purchased from Pierce (Rockford, IL, USA). The annexin V-FITC/PI (propidium iodide) kit was from Strong Biotech Corporation (Taipei, Taiwan). Hybond ECL transfer membrane and ECL Western blotting detection kits were obtained from Amersham Life Sciences (Amersham, UK).

### 4.2. Preparation of 10-Acetylirciformonin B (10AB) Stock Solution

10-Acetylirciformonin B was isolated and purified from the marine sponge, *Ircinia* sp. and its chemical structure was identified by the interpretation of its spectral data (1H-NMR, 13C-NMR, and 2D NMR), as previously described [23]. This compound was dissolved in DMSO at a concentration of 6 μM and diluted before used.

### 4.3. MTT Proliferation Assay

Cells were seeded at 4 × 10^4^ per well in 96-well culture plates before treatment with different concentrations of the tested compound [26]. After treatment for 24, 48, or 72 h, the cytotoxicity of the tested compound was determined using the MTT cell proliferation assay (thiazolyl blue tetrazolium bromide, Sigma-M2128). Light absorbance values (OD = OD_570_ − OD_620_) were recorded at 570 and 620 nm using an ELISA reader (AnthosLabtec Instrument, Salzburg, Austria)for calculating the concentration that caused 50% inhibition (IC_50_), *i.e.*, the cell concentration at which the light absorbance value of the experimental group is half that of the control group. These results were expressed as a percentage of the control ± SD established from *n* = 4 wells per experiment from three independent experiments.

### 4.4. Annexin V/PI Apoptosis Assay

The externalization of phosphatidylserine (PS) and membrane integrity were quantified using an annexin V-FITC staining kit [26]. In brief, 10^6^ cells were grown in 35 mm diameter plates and were labeled with annexin V-FITC (10 μg/mL) and PI (20 μg/mL) prior to harvesting. After labeling, all plates were washed with a binding buffer and harvested. Cells were resuspended in the binding buffer at a concentration of 2 × 10^5^ cells/mL before assessment on a FACS-Calibur flow cytometer (Beckman Coulter, Taipei, Taiwan) and analysis with CellQuest software. Approximately 10,000 cells were counted for each determination.

### 4.5. Determination of ROS Generation, and MMP Disruption

These assays were performed as described previously [26]. MMP disruption and ROS generation were detected with JC-1 cationic dye (5 μg/mL) and the carboxy derivative of fluorescein (carboxy-H_2_DCFDA, 1.0 mM), respectively. In brief, the treated cells were labeled with a specific fluorescent dye for 30 min. After labeling, cells were washed with PBS and resuspended in PBS at a concentration of 1 × 10^6^ cells/mL before analysis via flow cytometry.

### 4.6. Assay of Topoisomerase II Catalytic Inhibitors and Poisons

The assay was performed as described previously [25,26]. Standard relaxation reaction mixtures (20 μL) containing 50 mM Tris–HCl (pH 8.0), 10 mM MgCl_2_, 200 mM potassium glutamate, 10 mM dithiothreitol, 50 μg/mL bovine serum albumin, 1 mM ATP, 0.3 μg of pHOT1 plasmid DNA, two units of human topoisomerase II (Topogen, Columbus, OH, USA), and the indicated concentrations of etoposide and 10AB were incubated at 37 °C for 30 min. Reactions were terminated by adding 2 μL of 10% SDS to facilitate trapping the enzyme in a cleavage complex, followed by the addition of 2.5 μL of proteinase K (50 μg/mL) to digest the bound protein (incubated at 37 °C for 15 min) and finally by adding 0.1 volume of the sample loading dye. The DNA products were analyzed via electrophoresis through vertical 2% agarose gels at 2 voltages/cm in 0.5× TAE buffer. Gels were stained with ethidium bromide and photographed using an Eagle Eye II system (Stratagene, La Jolla, CA, USA).

### 4.7. Co-Immunoprecipitaion and Western Blotting

Cell lysates were prepared by treating the cells for 30 min in RIPA lysis buffer, 1% Nonidet P-40, 0.5% sodium deoxycholate, 0.1% sodium dodecyl sulfate (SDS), 1 mM sodium orthovanadate, 100 μg/mL phenylmethylsulfonyl fluoride, and 30 μg/mL aprotinin (all chemicals were obtained from Sigma Aldrich). The lysates were centrifuged at 20,000×*g* for 30 min, and the protein concentration in the supernatant was determined using a BCA protein assay kit (Pierce, Rockford, IL, USA). Proteins were immunoprecipitated with the indicated antibodies. The precleared protein A/G PLUS-agarose beads (Santa Cruz Biotechnology, Santa Cruz, CA, USA) were incubated with immunocomplexes and washed with the lysis buffer. Equal amounts of proteins were separated on 7.5%, 10% or 12% gels viaSDS-polyacrylamide gel electrophoresis and were subsequently electrotransferred to a PVDF membrane. The membrane was blocked with a solution containing 5% non-fat dry milk TBST buffer (20 mM Tris-HCl, pH 7.4, 150 mM NaCl, and 0.1% Tween 20) for 1 h and washed with TBST buffer. Protein expression was monitored by immunoblotting assay using specific antibodies. These proteins were detected by an enhanced chemiluminescence kit (Pierce). Quantitation of protein expression was performed using Image J software (National Institutes of Health, Bethesda, MD, USA).

### 4.8. Immunofluorescence Analysis

After treatment with 10AB, cells were stained with 200 μM of Mitotracker for 30 min, fixed with 4% paraformaldehyde in 50 mM HEPES buffer (pH 7.3) for 30 min, and permeabilized for 20 min with 0.2% Trition X-100 in PBS (pH 7.4). To prevent non-specific protein binding, cells were incubated with 5% BSA in PBS containing 0.05% Trition X-100 (T-PBS) for 1 h at room temperature. The cells were then incubated with the primary cytochrome *c* antibodies (1:250) for 2 h and further with secondary antibodies (Alexa Fluor 586-conjugated goat anti-mouse IgG (H + L), Life Technologies, Carlsbad, CA, USA) diluted at 1:1000 for 1 h at room temperature.After washing with PBS, cells were observed under a FV1000 confocal laser scanning microscope (Olympus, Tokyo, Japan).

### 4.9. Statistics

The results were expressed as means ± standard deviation (SD). Comparison in each experiment was performed using an unpaired Student’s *t*-test and a *p* value of less than 0.05 was considered to be statistically significant (* *p* < 0.05; ** *p* < 0.01; ** *p* < 0.001).

## 5. Conclusions

In the current study, we investigated the molecular targets of the cytotoxic furanoterpenoid derivative, 10AB, isolated from the marine sponge *Ircinia* sp. This furanoterpenoid derivative proved to be an interesting cytotoxic agent through its potent activity in several cancer cell lines with special selectivity for human acute myelocytic leukemia HL 60 cells [23,24]. Evaluation of the molecular targets of 10AB in HL 60 cells indicated that this compound suppressed topo IIαactivity and led to the accumulation of intracellular ROS followed by mitochondrial dysfunction. It also activated the expression of caspases and pro-apoptotic proteins. Moreover, it suppressed anti-apoptotic proteins, caspase inhibitors, XIAP, and survivin, which eventually led to apoptotic cell death. Scavenging ROS with NAC diminished the disruption of mitochondrial membrane potential and suppressed the apoptotic effect induced by 10AB treatment. Our results clearly suggested that the 10AB-induced mitochondrial apoptosis is directly mediated through ROS overproduction and mitochondrial dysfunction. These findings will provide opportunities for the future development of 10AB as a potential anti-cancer agent.
